# Overexpression of MiR-506 in Jurkat (Acute T Cell Leukemia) Cell Line

**DOI:** 10.30699/ijp.2020.119627.2298

**Published:** 2020-07-16

**Authors:** Shaghayegh Rostami Yasuj, Narges Obeidi, Gholamreza Khamisipou, Zeynab Gharehdaghi, Zivar Zangeneh

**Affiliations:** Department of Hematology, School of Para Medicine, Bushehr University of Medical Sciences, Bushehr, Iran

**Keywords:** Acute Lymphoid Leukemia, Apoptosis, Jurkat cell, MicroRNA, miR-506, p53 Genes

## Abstract

**Background & Objective:**

Acute lymphoblastic leukemia (ALL) is a malignant disease that arises from various mutations in B or T-lymphoid progenitors. MicroRNAs (miRNAs) regulate gene expression by binding to the 3' untranslated region of protein-coding genes. Dysregulation of miRNA expression may result in the development of cancerous phenotypes. Therefore, for the first time in this field, the present study aims to investigate the effect of overexpression of miR-506 in Jurkat (acute T cell leukemia) cell line.

**Methods:**

In this study, Jurkat cell lines were cultured in RPMI-1640 medium. Next, miR-506 was transfected with concentrations of 50 and 100 nM with Lipofectamine 2000. The accuracy of the transfection was confirmed by the transfection of siRNA conjugated with FITC. 48 h after transfection, the cells were prepared for other tests (flow cytometry, MTT assay, and RNA extraction). The expression level of miR-506 in the cells was analyzed using the quantitative real-time reverse transcription polymerase chain reaction (qRT-PCR). Finally, SPSS 21 software was used for the data analysis.

**Results:**

According to our results, the viability of cells in concentrations of 50 and 100 nM was significantly higher than the control group. By overexpression of miR-506, the expressions of pro-apoptotic genes (*p53*, *p21*) and anti-apoptotic gene B-cell lymphoma-2 (*BCL-2*) are decreased and increased, respectively.

**Conclusion:**

This study showed that miR-506 may function as an oncogenic miRNA in the T- ALL cell line. In conclusion, overexpression of miR-506 leads to an increase in viable cancer cells.

## Introduction

Acute lymphoblastic leukemia (ALL) is a malignant disease that arises from various genetic mutations in B or T-lymphoid progenitors, leading to blast cell proliferation, and accumulation of leukemic cells in bone marrow (1). Tcell ALL (TALL) is a subtype of ALL based on immunophenotype. It accounts for ~12% and ~25% of cases of ALL in children and adult patients, respectively (2). In general, patients with TALL have a poorer prognosis than other ALL patients, including ~15% and ~50% of pediatric and adult cases respectively, which indicates resistance to treatment or the risk of experiencing relapse (2, 3). Therefore, in order to improve the prognosis of patients with ALL and increase the efficacy of current treatments while reducing their side effects, identification of the molecular mechanisms is vital (4).

 Finding new therapeutic targets like microRNAs (miRNAs) is appealing and worth investigating. MiRNAs are 18–25 nucleotide, single-stranded non-coding RNAs that mediate the expression of various genes (5, 6). They regulate gene expression by binding directly to the 3' untranslated region (3'UTR) of protein-coding genes (7, 8). It is estimated that approximately 50% of all protein-coding genes are regulated by miRNAs. Also, these small molecules play critical roles in different types of biological cell processes, including cell proliferation and differentiation, organ development, apoptosis, and metabolism (9).

However, some miRNAs act as oncogenes in cancers by negatively regulating tumor suppressor genes (10). Among these targets, the tumor suppressor genes *p53* and *p21* are particularly important, since they can be a direct target of miRNAs and regulate the expression and processing of them (11, 12). It has been shown that these tumor suppressors can be inactivated by miRNAs overexpression in malignancies and may result in the development of cancerous phenotypes (12, 13). Therefore, it is important to identify dysregulated miRNAs and their targets for the detection of cancers and finding new therapeutic strategies (10).

In T-ALL like other cancers miRNAs can be dysregulated (14). Sayadi *et al.* showed that the overexpression of miR-192 in ALL patients upregulated tumor suppressor gene *p53* and downregulated prosurvival gene *BCL2*, which in turn induced apoptosis (15). Mavrakis *et al.* identified five miRNAs (miR-19b, miR-20a, miR-26a, miR-92, and miR-223) that could promote T-ALL development in a mouse model. They found that these miRNAs increased the pathogenesis of T-ALL by targeting tumor suppressor genes (16). Moreover, in another study, it was shown that four miRNAs (miR-101, miR-140-5p, miR-448, and miR-485-5p) were downregulated in T-ALL patient specimens and cell lines (17). However, for better diagnosis and treatment of T-ALL patients, other leukemic oncogenic miRNAs are required to be identified.

MiR-506 is considered as a component of an X chromosome-linked miRNA cluster in the primate testis (18, 19). Depending on the tumor types, miR-506 has reportedly functioned as a tumor-suppressive or an oncogenic miRNA (20). Moreover, previous studies have focused on the role of miR-506 in solid tumors like ovarian cancer (21), pancreatic cancer (22) and cervical cancer (18). However, the biological function of miR506 in TALL cells remains unclear.

This study aimed to investigate the effects of miR506 on the proliferation and viability of the TALL cell line (Jurkat). In addition, to examine the molecular mechanism of miR-506, the effect of this miRNA on *p53* pathway genes like *p21*, p53, and prosurvival B-cell lymphoma-2 (*BCL-2*) has been investigated by quantitative real-time polymerase chain reaction (qRT-PCR).

##  Materials and Methods

**Cell Line and Cell Culture**

The human T-ALL cell line Jurkat was obtained from the Iranian Stem Cell Technology Research Center (Tehran, Iran), and cultured in RPMI-1640 medium with L-glutamine (Cell Biotechnology Saba Arna, Fars, Iran), with 10% fetal bovine serum (FBS) (Gibco, USA), 100 U/mL penicillin and 100 mg/mL streptomycin. Then, the cell was incubated at 37°C under a humidified atmosphere consisting of 95% air and 5% CO_2_. Finally, suspension-cultured cells were used for transfection at 80% conﬂuence (6).

**Transfection**

The Jurkat cell lines were divided into control group, scramble group, as well as two treated groups that were transfected by 50 and 100 nM of miR-506 (Eurofins Genomics Germany). The sequence of the mature miR-506 was as follows: (5'-UAAGGCACCCUUCUGAGUAGA3'). For Jurkat cells transfection, Lipofectamine 2000 (Invitrogen, USA) was used according to the manufacturer’s manual. Briefly, firstly, one day before the transfection, Jurkat cells were seeded in 24-well plates at a concentration of 2×10^5^ cells/well in a medium with serum but without antibiotics. Secondly, 50 and 100 nM miR-506 were diluted in 50 μL OptiMEM in two tubes (Gibco, USA). Thirdly, in two other tubes, 1.5 μL Lipofectamine was diluted in 50 μL OptiMEM in each. After 5 min of incubation, the diluted miR-506 from stage 2 was combined with the diluted Lipofectamine from stage 3. Then, the compound was incubated at room temperature for 20 min. Finally, it was added directly to each well-containing cells (from stage 1) and was mixed gently. Non-transfected Jurkat cell line was considered as the control group. siRNA conjugated with FITC (Santa Cruz Biotechnology, Inc, Texas, USA) was utilized as a scramble and transfected at a final concentration of 50 nM in a similar manner. Transfection efficiency was detected by scramble and qRT-PCR. 

**MTT Assay**

The day after transfection, fresh RPMI containing 10% FBS replaced the culture medium. The culture plates were incubated at 37°C for the following day in a humidified atmosphere containing 5% CO_2_. Then, 10 μL 3-[4, 5-dimethylthiazol-2-yl]-2, 5 diphenyl tetrazolium bromide (MTT) solution (5 mg/mL) was added to each well and the plates were incubated for a further 4 h. After removal of the medium, dimethyl sulfoxide (DMSO) was added to each well (23). The culture plates were shaken for 15 min and the absorption was measured at the wavelength of 570 nm by an enzyme-labeled analyzer. Three independent experiments were performed. 

**Flow Cytometry Analysis**

Cell apoptosis was evaluated using an Annexin V-FITC Apoptosis Detection kit with 7-AAD (BioLegend, San Diego, CA, USA) (48 h after transfection) according to the following manufacturer’s manual (24): First, cells were washed with cold BioLegend's cell staining buffer and were resuspended in binding buffer. Subsequently, cells were incubated with 5 μL Annexin V/FITC and 5 μL 7-AAD for 15 min at room temperature in the dark. Then, 400 µL of Annexin V binding buffer was added to each tube of the cell. A flow cytometer (BD Biosciences) was used to detect apoptosis in Jurkat cells. Three independent experiments were performed. 

**RNA Extraction and** **Quantitative Real-Time Reverse Transcription Polymerase Chain Reaction (qRT-PCR) **

Total RNA was extracted from cultured cells in the different groups by the Total RNA Isolation Kit (Santa Cruz Biotechnology, Inc., Texas, USA), according to the manufacturer’s instruction, 48 h after the transfection. Nanodrop spectrophotometer (Thermo Fisher Scientific, Inc.) and electrophoresis on 2% agarose gel were used to determine RNA concentration after extraction (25). According to the manufacturer's instructions, cDNA of miRNA and other genes were generated using a highly sensitive cDNA Synthesis kit (Bonbiotech, Inc, Iran) and H Minus First Strand cDNA Synthesis Revert Aid kit (Thermo Fisher Scientifc, Inc., USA) respectively. Specific primers for PCR amplification were synthesized by Bonbiotech Company (Bonbiotech, Inc, Iran) as represented in Table 1.

 The expression level of miR-506 and mRNA in the cells were evaluated on the Step One Plus Real-Time PCR systems (ABI, US). Expression of *p53*, *p21*, and *BCL-2* mRNA in Jurkat cells were quantified using a 2X Real MOD Green PCR Master Mix kit (Takara Bio, Inc., Otsu, Japan), according to the manufacturer's instructions. Briefly, 2 µL cDNA product was diluted in a final volume of 20 µL, containing 10 pmol of each primer, 10 µL 2× reaction mixture of SYBR Green and 7.4 µL sterile deionized water (12). The cycling program was as follows: An initial denaturation step at 95°C for 10 min, followed by 40 cycles including a denaturation step at 95°C for 10 sec, annealing and extension at 55°C for 40 sec. Hypoxanthine-guanine phosphoribosyl transferase (HGPRT) and U6 snRNA were selected as internal housekeeping controls. All PCRs were performed in triplicate. The relative expression levels of miR-506 and *p53*, *p21*, and *BCL-2* mRNA were calculated by the 2^-ΔΔCT ^method (26).

**Statistical Analysis**

All statistical analyses were performed using SPSS 21.0 software (SPSS, Inc., Chicago, IL, USA). The data are presented as the mean ± standard deviation (SD) from at least three independent experiments. In order to make a general comparison of the difference between treated cell groups and the control group, the Kruskal-Wallis test was utilized. The difference among the groups (two by two) was analyzed by the Mann-Whitney test. P-value<0.05 was considered to indicate a statistically significant difference.

## Results

**Accuracy of Transfection**

**Fig. 1:**
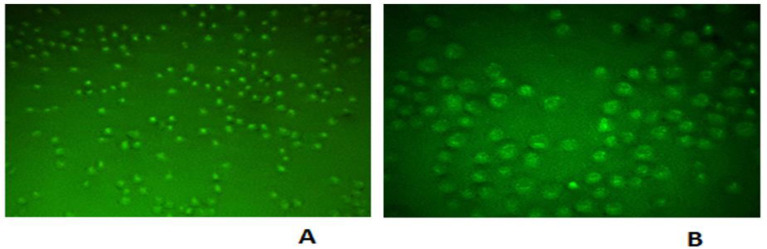
Green light of FITC attached to siRNA in the scramble sample. It indicates the correctness of the Transfection. A (10x magnification), B (40x magnification).

Following the transfection (Six hours), the scramble group (absorbance: 490 nm, emission: 514 nm) was examined by the green filter of the fluorescent microscope. Observing inside the cells through the green light of the fluorescent, the accuracy of transfection was confirmed (Figure 1).

**Table 1:** Sequence of Real Time PCR primers

**Primer Sequence**	Direction	**Gene**
**5'-** ** TAAGGCACCCTTCTGAGTAGA** ** -3'**	F	MicroRNA-506
**5'-** ** GCGAGCACAGAATTAATACGAC** ** -3'**	R	
**5'-CTCGCTTCGGCAGCACATATAC-3'**	F	U6 small nuclear RNA
**5'-ACGCTTCACGAATTTGCGTGTC-3'**	R	
**5'-TAACAGTTCCTGCATGGGCGGC-3'**	F	*p53*
**5'-AGGACAGGCACAAACACGCACC-3'**	R	
**5'-GAGGCCGGGATGAGTTGGGAGGAG-3'**	F	*p21*
**5'-CAGCCGGCGTTTGGAGTGGTAGAA-3'**	R	
**5'-GGTGGGGTCATGTGTGTGG-3'**	F	*BCL-2*
**5'-CGGTTCAGGTACTCAGTCATCC-3'**	R	
**5'-GGACAGGACTGAACGTCTTG-3'**	F	HGPRT
**5'-ATAGCCCCCCTTGAGCACAC-3**	R	
**HGPRT,** **Hypoxanthine-guanine phosphoribosyl transferase; BCL-2,** **B-cell lymphoma 2;** **F, forward; R, reverse**		

**MiR-506 Increased the Viability of Jurkat Cells**

**Fig. 2:**
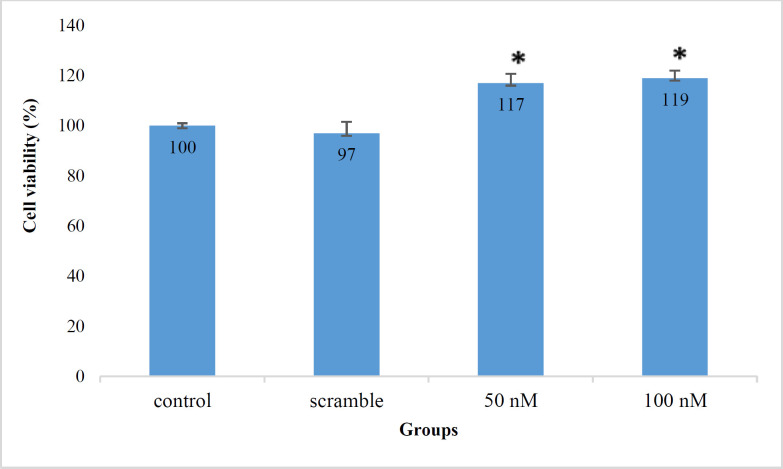
Viability of treated and non-treated groups with miR-506 based on MTT test (%).*P<0.05

Using MTT assays, we attempted to determine whether the viability of Jurkat cells was affected by miR-506. Our data indicated that the viability of Jurkat cells increased in comparison with the control group when the cells were transfected with 50 and 100 nM of miR-506) *P*=0.037, 0.037, respectively). Moreover, the viability of cells transfected with 100 nM of miR-506 increased in comparison with 50 nM group, but the difference was insignificant (*P*=0.37) and survival of the scramble group was not significantly different from the control group (*P*=0.48(. The viability mean of the studied groups with triple repetition is represented in Table 2 and Figure 2.

**Table 2:** Viability of Jurkat cells by MTT method (%)

**Treated**		**Non treated**
**100nM**	50nM	Scramble
**119±3.0**	117 ±3.7	97±4.5

**MiR-506 Decreased Apoptosis in Jurkat Cell**

**Fig. 3:**
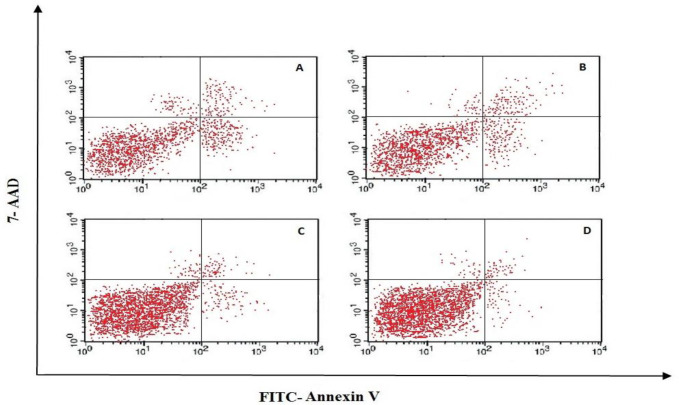
Flow cytometric results of treated and nontreated cell groups. A. Control group, B. Scramble, C. treated with 50 nM of miR-506, D. treated with 100 nM of miR-506

**Fig. 4:**
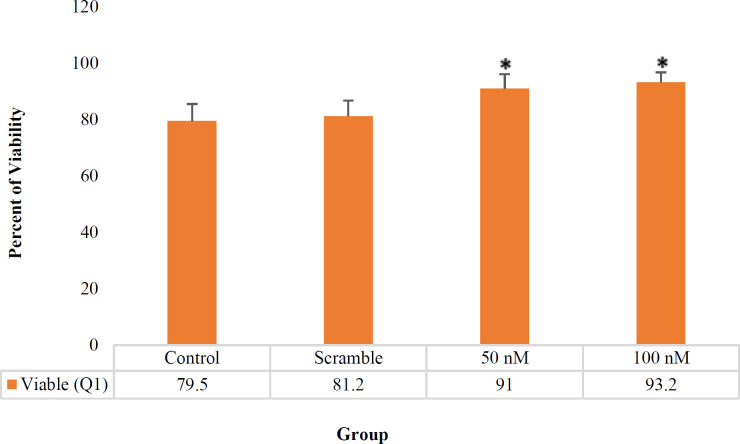
Viable cell Q1 in treated and non-treated groups (%). In transfected cells with miR-506, the viable cells increased significantly compared with the control group and the scramble. *P<0.05

For cell apoptosis assay, the treated and non-treated Jurkat cells were stained with Annexin V-FITC and 7-AAD and were analyzed by the flow cytometry. The position of viable, apoptotic, and necrotic cells in the flow cytometry quadrant (according to the type of absorbent stains) is illustrated in Table 3. The result showed that increasing miR-506 significantly inhibited the apoptosis of Jurkat cells. In other words, increasing the overexpression of miR-506 in transfected groups (50 and 100 nM) led to higher cell viability, compared to the control group (91%, 93.2%, 79.5%, respectively, as shown in Figures 3 and 4).

**Table 3:** The position of viable, apoptotic and necrotic cells in the flow cytometry quadrant according to the type of absorbent stain

**7-AAD**	**Annexin-V**	**stain**
		Cell Condition
**Neg**	Neg	Viable (Q1)
**Neg**	Pos	Early Apoptotic (Q2)
**Pos**	Pos	Late Apoptotic (Q3)
**Pos**	Neg	Necrotic (Q4)
		Pos; Positive, Neg; Negative

**Fig. 5:**
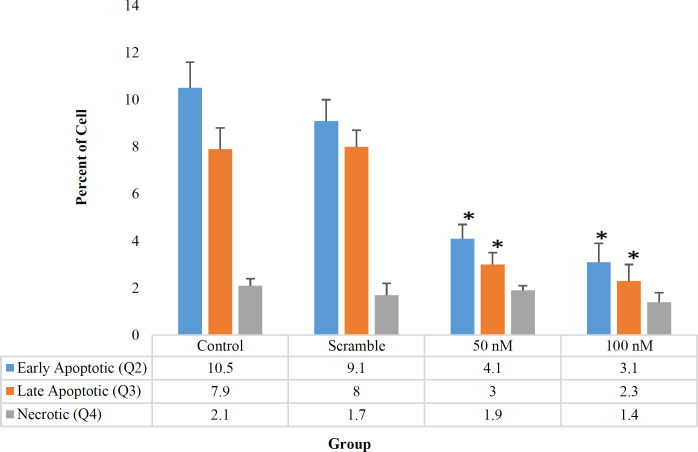
Percentage of primary, late apoptotic and necrotic cells in Q2, Q3, Q4. By increasing the transfection of miR-506, the total apoptotic cells decreased significantly compared to the control group and the scramble *P<0.05

The viable cells were significantly different in the treated group (50 and 100 nM) compared with the control group (*P*= 0.034, 0.022), but there was no significant difference between the treated groups (50 and 100 nM) (*P*=0.37). According to Figure 5, total apoptotic cells (early apoptotic plus late apoptotic) in the treated group (50 and 100 nm) were significantly different compared with control (*P*= 0.046, 0.038 respectively), while total apoptotic cells in treated groups (50 and 100 nm) didn’t show a significant difference in comparison with each other (*P*=0.37). Also, the percentage of necrotic cells in treated groups (50 and 100 nM) compared with the control group and also between the two treated groups were not significantly different (*P*= 0.09, 0.07, 0.08, respectively).

**Overexpression of miR-506 in Treated Groups**

**Fig. 6:**
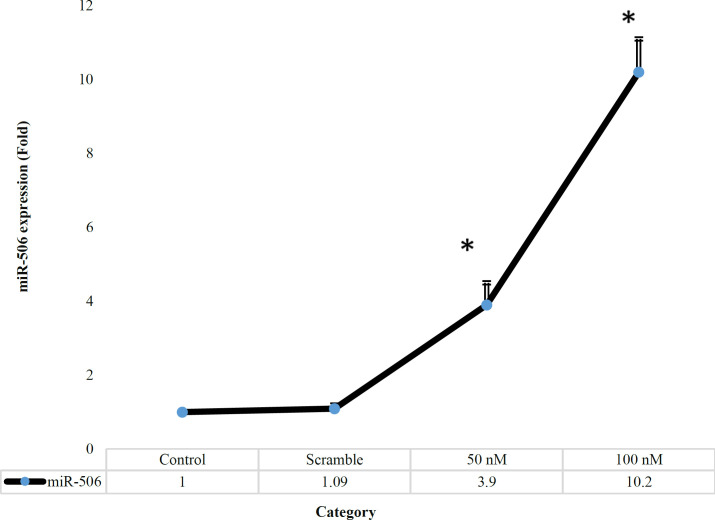
The expression of miR-506 in treated and non-treated cell groups. *P<0.05

For higher confirmation of the transfection of miR-506 in Jurkat cell, the expression of miR-506 was measured via qRT-PCR. As shown in Figure 6, the miR-506 expression in the control group was significantly different compared with 50 and 100 nM groups (*P*= 0.037, 0.037, respectively). In other words, in 50 nM and 100 nM groups, a fold change of 3.9 and 10.2 was observed, respectively, indicating a significant difference between 50 nM and 100 nM groups (*P*=0.04).

**P53 Gene Expression Reduced in Cells Transfected with miR-506**

**Fig. 7:**
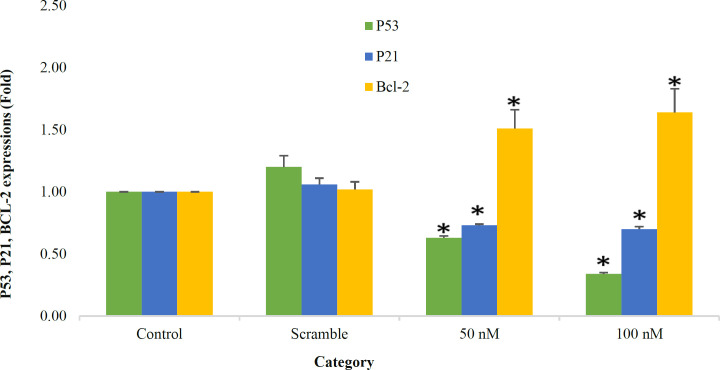
Expression of pro-apoptotic and anti-apoptotic genes in treated and non-treated cell groups *P<0.05

To further understand the role of miR506 in the apoptosis of Jurkat cells, the expression of *p53* as a regulatory gene of apoptosis was assessed by qRT-PCR. As represented in Table 4 and Figure 7, *p53* gene expression in groups transfected with miR-506, significantly downregulated (fold change of 0.63, 0.34 in 50 and 100 nM, respectively) compared with the control (*P*=0.037, 0.037). Moreover, the gene expression in the 50 nM group was remarkably higher than the 100 nM group (*P*=0.04). It was found that *p53* gene expression decreased following overexpression of miR506 

**P21 Gene Expression Reduced in Cells Transfected with miR-506**

The relation between *p21* expression and overexpression of miR-506 in two 50 and 100 nM concentrations in Jurkat cells, was assessed by qRT-PCR. As shown in Figure 7 and Table 4, *p21* gene expression in groups transfected with miR-506 was significantly downregulated compared with the control group (fold change in 50 and 100 nM: 0.90, 0.87, respectively; *P*=0.037 in each). No significant difference was shown between 50 and 100 nM groups (*P*=0.09).

**BCL2 gene expression increased following overexpression of miR506**

In order to evaluate whether miR-506 regulated BCL2, 48 h after transfection in Jurkat cells, BCL2 mRNA levels were measured via qRT-PCR. BCL-2 expression significantly increased in the treated group (50 and 100 nM) compared with the control (*P*= 0.034, 0.038). As demonstrated in Table 4 and Figure 7, BCL-2 gene expression increased after enhancing the dose of miR-506 (fold change in 50 and 100 nM: 1.51, 1.64, respectively). However, no significant difference was shown between the treated groups (*P*=0.06).

**Table 4:** Expression of miR-506, pro-apoptotic genes and anti-apoptotic genes in cell groups

Gene	miR-506	*p53*	*p21*	*BCL-2*
**GROUP**				
**Sramble**	1.09±0.100	1.20±0.090	1.06±0.050	1.02±0.060
**50 nM**	3.90±0.006	0.63±0.020	0.7**3**±0.010	1.51±0.150
**100 nM**	10.20±0.900	0.34±0.010	0.7**0**±0.015	1.64±0.190

Data are presented as the mean of fold changes ± standard deviation. *BCL-2*, B-cell lymphoma

2; miR, microRNA

## Discussion

Many studies have shown that dysregulation of miRNAs expression can lead to tumor progression and initiation. Therefore, the identification of dysregulated miRNAs in the pathogenesis of malignancy may help in finding more effective cancer therapy (4). Recently, the aberrant expression of miR-506 in different types of malignancies has been identified. For example, Banales *et al.* found that miR-506 upregulated in cholangiocytes of patients with Primary Biliary Cirrhosis which led to impaired function of Cl^−^/HCO_3_^−^anion exchanger 2 (AE2) and an increase in Primary Biliary Cirrhosis (27). Streicher *et al.* demonstrated that the miR-506-514 cluster was significantly overexpressed in melanoma tissue and promoted melanoma growth (28). In addition, Ananthanarayanan *et al.* discovered that miR-506 downregulated InsP3R3 expression and impaired Ca^2+^ signaling and secretion in duct cells (cholangiocytes) thus, inhibited fluid secretion (29). In contrast, this miRNA acts as a tumor suppressor in colorectal, breast, and ovarian cancers, and inhibits tumor proliferation (21, 30, 31). In general, weather this miRNA functions as an oncogene or a tumor suppressor seems to be dependent on the cell type (32). However, the role of miR-506 in T-ALL progression remains unknown.

In the present study, to further investigate the effects of miR506 on TALL, Jurkat cells were transfected with miR506, which induced an increase in the expression of miR506. The overexpression of miR-506 could enhance the viability of Jurkat cells and decrease their apoptosis rate. These findings suggest that miR506 may have an oncogenic role in Jurkat cells. 

In previous studies, it has been demonstrated that miR506 might regulate different genes associated with various malignancy progressions, including YAP, ROCK1, CDK4/6-FOXM, IQGAP1 and SPHK1 (33). Moreover, another gene regulated by miR-506 is *p53* suppressor tumor, which plays a critical role in cancers. Song *et al.* reported that miR-506 was consistently overexpressed in retinoblastoma cells and tumor specimens. In addition, they showed that inhibition of miR-506 led to the upregulation of both *p53* and Bax and induced suppression of cell viability (33). In contrast, in another study, it was shown that Ectopic expression of miR-506 inhibited the NF-κB pathway and increased ROS generation, which in turn activated *p53* to suppress lung cancer cell viability (34). 

*p53* is a tumor suppressor/transcription factor that regulates various cell functions in humans and other high eukaryotes (35). In reaction to diverse stressors, *p53* transactivates many downstream genes, such as *p21* and PUMA. The interaction between *p53* and *p21* creates the p53/p21 complex, which binds to prosurvival *BCL-2* family proteins to liberate pro-apoptotic groups such as Bax and Bak, consequently promoting apoptosis or cycle arrest (13, 36). In many cancers, the functions of *p53* pathway components, such as *p21* and *p53*, have been disrupted. On the other hand, many studies have shown that miRNAs may target components of this pathway and potentially inhibit their expression, thereby increasing malignancies (13, 35). Moreover, *BCL-2* acts as an apoptosis regulator and has been shown to play a critical role in the pathogenesis of various types of cancer (12). 

Lv *et al.* demonstrated that overexpression of miR-149 significantly inhibited chondrocyte apoptosis by decreasing the protein levels of Bax and *p53* and increasing the levels of *BCL-2* (37). It has also been shown that miR-34a plays an anti-tumor role in human esophageal squamous cancer cells and inhibits cellular growth by downregulation of Sirtuin1 (SIRT1) and upregulation of p53/p21 (38). However, the effect of miR-506 on the components of the *p53* pathway in T-ALL has not been investigated yet. 

In the present study, the results of qRT-PCR assay indicated that overexpression of miR506 in Jurkat cells resulted in an increase in the levels of prosurvival *BCL-2* mRNA. In addition, mechanistic investigations determined that miR-506 may enhance the viability of Jurkat cells partially by regulating and increasing the level of *BCL-2*. Moreover, this study has demonstrated that miR-506 overexpression is inversely correlated with lower *p53* and *p21* mRNA levels in Jurkat cells. Therefore, overexpression of miR-506 was associated with a decrease in the expression of *p53* and *p21* genes, which could lead to apoptosis reduction. Overall, increasing the expression of *BCL-2* and decreasing the expressions of *p53* and *p21*, may play an important role in oncogenic function of miR-506 in T-ALL.

## Conclusion

In conclusion, overexpression of miR-506 in Jurkat cell lines can promote cell viability via increasing *BCL-2* expression. Furthermore, it can reduce cell apoptosis by lowering the expression of *p53* and *p21*. Thus, our findings revealed the significant roles of miR-506 in T-ALL pathogenesis, and in consequence, prompting us to consider the therapeutics role of miRNAs against cancer. Last, additional studies are required to clarify the exact interaction mechanism between miRNAs and the *p53* pathway.
